# Effect of tactile stimulation on the level of consciousness and vital signs of patients with traumatic brain injury: a quasi- experimental study

**DOI:** 10.62838/jccm-2026-0032

**Published:** 2026-07-27

**Authors:** Richa Bhogal, Nimarta Rana, Nipin Kalal, Khina Sharma, Suryanarayanan Bhaskar

**Affiliations:** Department of Medical Surgical Nursing, College of Nursing, AIIMS Jodhpur, Rajasthan,India; College of Nursing, Dr RMLIMS Lucknow, Uttar Pradesh,India; Department of Neurosurgery, AIIMS Jodhpur, Rajasthan,India

**Keywords:** Traumatic brain injury, tactile stimulation, level of consciousness, vital signs

## Abstract

**Introduction:**

Patients with traumatic brain injury (TBI) are subjected to multiple stressors, including environmental changes, medical treatment, and surgical procedures. Hospitalization in an unfamiliar setting, especially when separated from family members, can increase anxiety and stress, which may adversely influence vital signs and levels of consciousness. Tactile stimulation, when integrated with routine medical management, may contribute to improving patient outcomes. Therefore, the present study aimed to determine the effect of tactile stimulation on the level of consciousness and vital signs among patients with traumatic brain injury.

**Materials and Methods:**

A quasi-experimental study was conducted in the neurosurgery wards and intensive care units of a tertiary care hospital. A total of 40 patients with mild (Glasgow Coma Scale [GCS] score 13–15) and moderate (GCS score 9–12) traumatic brain injury were selected using a consecutive sampling technique and equally allocated into experimental and control group (n = 20 each). The experimental group received tactile stimulation in addition to conservative care daily at 8 a.m., 12 pm, and 4 p.m. for three consecutive days, with each session lasting 15–20 minutes. The control group received only conservative care. Data were collected using a sociodemographic and clinical profile, a vital sign monitoring sheet, and the Glasgow Coma Scale. Statistical analysis was performed using the Chi-square test, Fisher's exact test, Friedman test, and Mann–Whitney U test.

**Results:**

Both groups were comparable at baseline, with no statistically significant differences in socio-demographic and most clinical variables. The majority of patients had a GCS score of 9–12 at admission, and road traffic accidents were the predominant cause of injury. A statistically significant difference was observed only in the type of brain injury (p < 0.001). No statistically significant differences were found between the groups in vital signs or GCS scores across day 1, day 2, and day 3 following the intervention, indicating similar clinical outcomes.

**Conclusion:**

The findings indicate that no statistically significant differences were observed between experimental and control groups, indicating that the intervention had no significant effect on the measured clinical outcomes. The limited duration of intervention may have constrained its effectiveness.

## Introduction

“Worldwide, TBI is among the leading causes of mortality, morbidity, and impairment” [1]. The majority of TBIs in India are attributed to road traffic accidents [2]. Among all TBI survivors, 70–90% experiences a mild TBI, while 10–30% experiences a moderate or severe TBI [3]. In India, a TBI-related death occurs every 6–10 minutes and it is 25 times more prevalent than in any other developed country [4].

The severity of the brain injury directly correlates with both, outcome at discharge and future quality of life. Patients with moderate to severe brain injury may experience sensory overload or deprivation during their hospital admission. Sensory changes can result from various causes such as brain damage, separation from family, sedatives use, prolonged ventilation, exposure to excessive and irrelevant hospital noise and undergoing multiple painful invasive procedures [5]. Patients with TBI who sustain an injury have a poor quality of life and frequently experience unconscious periods, during which they are unable to perceive or interact with their surroundings [6].

The most important aspect of nursing care for these patients is the prevention of loss of sensory functions [5]. One of the primary aims of evidence-based care in hospitals is to integrate innovative, cost-effective and efficient methods in conjunction with pharmacological and non-pharmacological interventions. Tactile stimulation is proposed as a complementary approach to support nervous system functions and improve the level of consciousness in patients with brain injuries while, also promoting feelings of safety, comfort and emotional well-being [6].

A study by Moattari et al. found that tactile stimulation could increase a patient's level of consciousness, if it is provided within the first 72 hours of the brain injury [7]. A study conducted by Refaat et al. found that organized stimulation provided by family members increased the level of consciousness among comatose patients with traumatic brain injury, reduced the incidence of physiological events, and shorten the duration of the need to stay in hospital intensive care units.

A study conducted by Megha M. et al. in India found that short sessions of high frequency stimulation appear to be more beneficial. This exemplifies that administering multimodal stimulation five times daily for 20 minutes is better than administering it twice daily for 50 minutes [2].

Earlier research suggests that tactile stimulation can be helpful for patients with severe brain injuries (with GCS score <8); however, there is still limited information about whether this intervention is effective in improving consciousness among patients with mild to moderate brain injuries. In addition, most of the previous studies recorded that stimulation was provided by the family members rather than the trained nursing professionals [9]. Considering the need to prevent sensory deprivation and reduce the risk of long-term complications in patients with TBI, the present study aims to assess the effect of tactile stimulation on patient's level of consciousness as well as their vital signs.

## Materials and methods

### Study design and setting

A quasi-experimental, time series non-equivalent control group design was adopted for this study. Data was collected from the next of kin for patients with TBI admitted in the neurosurgery ward and ICUs of a tertiary care hospital. Patients were recruited using Consecutive sampling technique based on the following eligibility criteria: i) Patients aged between 18–65 years, ii) Patients with mild (GCS >13) and moderate (GCS between 9–12) brain injury, iii) Those with any type of TBI, including epidural, subdural, and subarachnoid haemorrhage, contusions and mixed types injuries, iv). Those with a Richmond agitation sedation score between −2 to +2.

The sample size was calculated using the paired data formula for mean:

n=[Z(1−α/2)+Z1−β]2/Δ2+Z(1−α/2)/2

μ1= The pre- intervention mean score for the experimental group upon initial admission; μ2= The post- intervention mean score for the experimental group on the 7th day, σ = Standard deviation of the experimental group

Where, Δ = μ1- μ2/σ: Z(1-α/2) = 1.96, Z1- β= 0.842

Δ = 7.15 − 6.10 / 1.52 = 0.6907
(1.96 + 0.842) 2/(0.69079) 2 + (1.96)/2
18.3726 =18
n = 18

The calculated sample size was 18 per group, yielding a total sample size of 36. Using a 10% dropout rate, the total sample size was determined to be 40 (n = 20 in each group).

### Statistical analysis

Data collected in this study was analysed using; IBM SPSS version 20. The Shapiro–Wilk test was performed to assess the normality of the data collected, which indicated that it was a non-normal distribution. Therefore, non-parametric statistical tests were applied. Descriptive statistics such as; frequency, percentage, median, interquartile range and the mean rank, were used to summarize sociodemographic and clinical variables. The Friedman test and Mann–Whitney U test were conducted to examine the relationship between vital signs and level of consciousness in patients with TBI. A significance level of 0.05 was considered for all analysis.

**Table:** Description of the intervention provided to the experimental and control group

**Group**		**Description**
**Experimental**	**Control**	
Step-1 Enrolment	Step-1 Enrolment	Before starting the intervention, informed and obtained signed consent from the patient's next of kin. Later, patients were recruited into an experimental (n=20) and a control group (n=20).
Step-2 Sociodemographic and clinical variables	Step-2 Sociodemographic and clinical variables	The researcher collected data related to sociodemographic and clinical variables before starting the intervention.
Step-3 Assessment of vital signs and level of consciousness.	Step-3 Assessment of vital signs and level of consciousness.	Vital signs and GCS scores were measured five minutes prior to each session over a period of three consecutive days.
Step-4 Intervention (Tactile Stimulation)	Step-4 Conservative care	Tactile stimulation was provided to patients with mild to moderate TBI, three times a day at 8am, 12pm and 4pm, for three consecutive days, with each session lasting between 15–20 minutes. It was carried out in three steps, with a 1-minute gap between each. Firstly; the hand surfaces were touched from the wrist down. Next, the shoulders and hands of the patients were touched with a soft brush. Lastly, a cotton-wisp wool applicator was used to touch the hands and forearm of patients.
Step-5 Reassessment of vital signs and level of consciousness	Step-5 Reassessment of vital signs and level of consciousness	Vital signs and GCS scores were reassessed again five minutes after each session to examine the effects of tactile stimulation.

### Ethical approval and informed consent

The institutional ethical committee granted the approval of the study vide certificate (reference no. AIIMS/IEC/2022/4056). Because the patients have impaired consciousness, the next of kin were informed about the nature and purpose of the study and signed consent was obtained from them.

## Results

A total of 40 patients admitted in neurosurgery ward and ICUs were included in the analysis. Baseline characteristics of patients are shown in Table I. Among all patients, most of the participants were males (85% in experimental group and 90% in control group). In addition, 50% of patients in the control group and 60% in the experimental group were accompanied by one family member in the hospital.

**Table 1:** Socio-demographic variables of TBI patients (N=40)

**Variable**	**Control Group (N=20) Freq. (%)**	**Experimental Group (N=20) Freq. (%)**	**Total (N=40) Freq. (%)**	**p value**
**Age (in yrs)**				
18–34	10(50)	10(50)	20(50)	0.189
35–50	8(40)	4(20)	12(30)	
51–65	2(10)	6(30)	8(20)	
**Gender**				
Male	18(90)	17(85)	35(87.5)	0.633
Female	2(10)	3(15)	5(12.5)	
**Marital status**				
Married	11(55)	17(85)	28(70)	0.084
Unmarried	9(45)	3(15)	12(30)	
**Education**				
No education	3(15)	4(20)	7(17.5)	
Primary & Secondary	15(75)	15(75)	30(75)	0.894
Graduation	2(10)	1(5)	3(7.5)	
**Occupation**				
Homemaker	3(15)	5(25)	8(20)	
Private job	11(55)	10(50)	21(52.5)	0.710
Govt. job	1(5)	2(10)	3(7.5)	
Unemployed	5(25)	3(15)	8(20)	
**Religion**				
Hindu	19(95)	18(90)	37(92.5)	0.486
Muslim	1(5)	2(10)	3(7.5)	
**Monthly income**				
>50,000	5(25)	9(45)	14(35)	0.185
<50,000	15(75)	11(55)	26(65)	
**Family type**				
Nuclear	4(20)	5(25)	9(22.5)	0.705
Joint	16(80)	15(75)	31(77.5)	
**Accompanying member**				
One member	11(55)	12(60)	23(57.5)	0.749
Two members	9(45)	8(40)	17(42.5)	
**Time spent by family member with patient/day**				
0–12hrs	4(20)	5(25)	9(22.5)	0.705
12–24hrs	16(80)	15(75)	31(77.5)	

* Significance Level p<0.05 (Chi square test and Fisher's exact test)

The results of chi-square and fisher exact test revealed that both groups were homogeneous, with no statistically significant differences in socio-demographic variables.

“The clinical variables of TBI patients included in this study are presented in Table II. Accordingly, the majority of patients in the control group (55%) and the experimental group (45%) had a Glasgow Coma Score of 9–12 at admission. Nearly 80% of patients in the control group and 85% in the experimental group were hospitalized for road traffic accidents. Nearly 3/4^th^, (75%) of patients in the control group were admitted with mixed-type injuries, whereas half of the patients (50%) in the experimental group were admitted with contusions. Just under half (45%) of patients in the experimental group and 45% in the control group received conservative care. There were no statistically significant differences in clinical variables, except for type of brain injury (p<0.001), which was statistically significant.

**Table 2:** Clinical variables of TBI patients (N=40)

**Variable**	**Control Group (N=20)** **Freq. (%)**	**Experimental Group (n=20)** **Freq. (%)**	**Total (n=40)** **Freq. (%)**	**p value**
**GCS at admission**				
Moderate injury (9–12)	11(55)	9(45)	20(50)	0.595
Mild injury (13–15)	9(45)	11(55)	20(50)	
**Cause of hospitalization**				
RTA	16(80)	17(85)	33(82.5)	0.677
Falls	4(20)	3(15)	7(17.5)	
**Type of injury**				
SDH	2(10)	4(20)	6(15)	
EDH	2(10)	3(15)	5(12.5)	0.001*
Contusions	1(5)	10(50)	11(27.5)	
Mixed	15(75)	3(15)	18(45)	
**Accompanying injuries**				
No injuries	19(95)	17(85)	36(90)	0.486
Limb fracture	1(5)	3 (15)	4(10)	
**Co morbidities**				
No	20(100)	17(85)	37(92.5)	0.231
Hypertension	-	3(15)	3(7.5)	
**Previous history of head injury**				
Yes	1(5)	2(10)	3(7.5)	0.548
No	19(95)	18(90)	37(92.5)	
**History of hospitalization in ICU**				
Yes	4(20)	6(30)	10(25)	
No	16(80)	14(70)	30(75)	0.465
**Medical Treatment**				
Antipyretics (PCM)	14(70)	17(85)	31(77.5)	
Diuretics (Mannitol)	3(15)	2(10)	5(12.5)	0.475
Antiseizures	3(15)	1(5)	4(10)	
**Management**				
Conservative	9(45)	10(50)	19(47.5)	
Craniotomy	4(20)	1(5)	5(12.5)	
Craniectomy	4(20)	4(20)	8(20)	0.641
Cranioplasty	2(10)	4(20)	6(15)	
Hemicraniectomy	1(5)	1(5)	2(5)	

GCS- Glasgow Coma Scale; RTA- Road Traffic Accident; SDH- Subdural Haemorrhage; EDH- Extradural Haemorrhage

* Significance Level (p<0.05), Chi- square test and Fisher's exact test

Table III displayed the between group comparison of vital signs and GCS. The results demonstrated that there were no statistically significant differences in vital signs (blood pressure, temperature, pulse, respiration and SPO2), and GCS on day 1, day 2 and day 3 following the intervention.

**Table 3:** Between group comparison of vital signs and GCS on Day1, Day2 and Day3 (N=40)

**Variable**	**Baseline**		**p value**	**After Intervention**		**p value**
	**Control Group (n=20) Mean Rank**	**Exp. Group (n=20) Mean Rank**		**Control Group (n=20) Mean Rank**	**Exp. Group (n=20) Mean Rank**	
**SBP**						
Day1	21.33	19.68	0.654	22.73	18.28	0.228
Day2	19.53	21.48	0.597	17.73	23.25	0.136
Day3	19.25	21.45	0.606	19.68	21.33	0.655
**DBP**						
Day1	22.00	19.00	0.417	20.65	20.35	0.935
Day2	21.33	19.68	0.655	21.05	19.95	0.777
Day3	19.50	21.50	0.587	20.10	20.90	0.888
**TEMP**						
Day1	20.28	20.73	0.890	20.65	21.00	0.747
Day2	19.00	22.00	0.317	21.05	22.15	0.370
Day3	19.00	22.00	0.262	20.10	23.68	0.85
**PULSE**						
Day1	22.28	18.73	0.336	22.15	18.85	0.370
Day2	23.28	17.73	0.133	23.13	17.73	0.133
Day3	23.38	17.64	0.119	23.68	17.33	0.085
**RESP**						
Day1	23.68	17.34	0.072	23.80	17.20	0.61
Day2	22.15	18.85	0.340	23.13	17.88	0.155
Day3	23.40	17.60	0.95	21.80	19.20	0.455
**SPO2**						
Day1	21.43	19.58	0.594	19.98	21.03	0.762
Day2	22.08	18.93	0.370	17.75	23.25	0.136
Day3	20.20	20.20	0.862	20.20	21.33	0.133
**GCS**						
Day1	21.25	19.75	0.676	21.60	19.40	0.541
Day2	21.35	19.65	0.635	20.78	20.23	0.879
Day 3	20.03	20.98	0.793	18.58	22.43	0.290

SBP- Systolic Blood Pressure; DBP- Diastolic Blood Pressure, GCS- Glasgow Coma Scale*Significance Level p<0.05 (Mann- Whitney U Test)**

## Discussion

The study results revealed; that tactile stimulation did not improve the vital signs and consciousness level of TBI patients. Our hypothesis was confirmed by the findings of the present study.

The current study results exhibited that the majority of patients were in the age group of 18–34 years and the majority of the patients were male in both the groups. These findings are consistent with a study by Ahmed R.F et al showing that the same findings aligned with our present study [9]. There were no statistically significant differences observed in socio-demographic variables between the control group and experimental group. These findings are exactly similar to those of studies conducted by Salmani F et al. and Othman Y.S et al [11]. The results revealed that most of the patients had moderate injuries (9–12 GCS) in the control group and mild injuries (13–15 GCS) in the experimental group. In both groups, majority of the patients were managed conservatively. In the present study, there were no statistically significant differences in clinical variables, except the variable type of injury that was statistically significant (p< 0.001). These findings are comparable with the other study results, Sedghi and Lynn et al. that found a statistically significant differences in clinical variable (type of injury) between the two group [6,18].

According to the results obtained by present study, there were no statistically significant differences observed in systolic and diastolic blood pressure between both the groups. These findings are contradictory with Fakhr Mohavedi et al. who added to recent evidence that a statistically significant difference was seen in systolic blood pressure after tactile stimulation in mechanically ventilated patients, whereas no significant differences were observed in diastolic blood pressure between the two groups [12]. Zare et al. emphasized that systolic and diastolic blood pressure were unaffected by sensory stimulation. These results coincide with those of the present research findings; this is because the intervention was provided for shorter time period [13].

Distinctive findings on temperature in current study revealed that there were no statistically significant differences between both the groups. There are other studies in the literature that showed the same findings as that of our study. A research study by Yekefellah et al. showed no differences in the mean score of temperature between both the groups as the intervention was provided for five minutes twice daily for five days [14]. Moreover, these study results are somewhere in line with the results noticed in our study. Fakhr Mohavedi et al. findings revealed that following tactile stimulation, the mean heart rate considerably dropped by four beats per minute than the baseline heart rate; therefore, these findings are not in concordance with present study's results [12].

The current study found that there was no significant decrease in the pulse rate of TBI patients between both the groups (p=0.085). The current study findings showed no statistically significant differences in respiratory rate between the both groups over the three days of stimulation (p=0.455). On the contrary Lakie et al. concluded that tactile stimulation improved respiratory status (p<0.001) of TBI patients, but these findings are not in resemblance with the findings obtained from our study [15]. Yousefi et al. study results displayed significant changes in oxygen saturation level of patients (p<0.001), whereas present study findings did not show any significant differences in SPO2 level of patients in both the groups (p=0.133) [16]. Lakie et al. in their study concluded that tactile stimulation improved the oxygen saturation level of patients with a higher statistically significant result (p<0.000). These findings contradict the results yielded from our study. Maleki et al. study findings mentioned that there were no statistically significant differences observed in the oxygen saturation level of TBI patients between the both groups [17]. Interestingly, these results supported the findings of our study.

Likewise, our study found that there were no statistically significant differences seen in GCS scores between both the groups and these findings of our study contradict Seo et al. which revealed a significant improvement in patients' consciousness levels after two weeks of intervention (p<0.001). We cannot predict that whether this improvement in the consciousness level of TBI patients would be maintained for the long term or not, this outcome generally depends on the duration of intervention provided to the TBI patients.

## Limitations

1. The quasi-experimental, non-equivalent control group design carries an inherent risk of selection bias and limits causal inference.
2. Differences in injury type between the groups may have affected neurological recovery and physiological stability. As this variable was not controlled during the analysis, it may have acted as a confounding factor.
3. Although nonparametric tests (Friedman, Mann–Whitney) were used, no multivariable or mixed-effects analyses were performed to control for important covariates such as injury type, baseline GCS, sedation/analgesia, surgical interventions, or other ICU management factors. This limits the robustness of the findings.
4. Intervention was provided for shorter duration in this study. So, this time period is not sufficient to observe significant neurological changes in patients with traumatic brain injury.
5. Blinding of outcome assessors was not implemented, which may introduce measurement bias, particularly in subjective assessments such as the Glasgow Coma Scale (GCS).
6. Variations in routine ICU care could potentially influence patient outcomes. Although patients in both groups received standard conservative management, detailed standardization of aspects such as sedation, intracranial pressure management, medications, and nursing care was not fully controlled.
7. The single-center design and small sample limit generalizability

## Conclusion

Based on the findings of this study, three days of tactile stimulation did not seem to have any discernible effect on TBI patient's vital signs and the level of consciousness between both the groups. Further research with longer or more intensive interventions may be required to adequately assess its effectiveness, as the short length of the intervention may have reduced its potential effects.
